# Foundation doctors’ induction experiences

**DOI:** 10.1186/s12909-015-0395-1

**Published:** 2015-07-24

**Authors:** Susan Miles, Joanne Kellett, Sam J. Leinster

**Affiliations:** 1 Norwich Medical School, University of East Anglia, Norwich, UK; 2 Norfolk and Norwich University Hospital, Clinical Research and Trials Unit, Norwich Medical School, University of East Anglia, Norwich, UK

**Keywords:** Induction, Trainee doctor, Foundation programme, Preparedness for practice, Transition, Medical education, Postgraduate training

## Abstract

**Background:**

It is well established that trainee doctors struggle with the transition from medical school to starting work and feel unprepared for many aspects of their new role. There is evidence that suitable induction experiences improve competence and confidence, but available data indicate that trainee doctors on the UK Foundation Programme are commonly not experiencing useful inductions. The aim of the reported research was to explore trainee doctors’ experiences with induction during their first year of the Foundation Programme to identify the most useful characteristics.

**Methods:**

A questionnaire was designed to explore trainee doctors’ experiences with induction at two points during their first Foundation year, during the first and third of three rotations, to enable all induction experiences on offer during the year to be surveyed. Data were collected using an anonymous questionnaire distributed during a teaching session, with an online version available for those trainees not present. Questions gathered information about characteristics of the inductions, usefulness of components of the inductions and what gaps exist.

**Results:**

192 Foundation trainee doctors completed the questionnaire during Rotation 1 and 165 during Rotation 3. The findings indicated that induction experiences at the beginning of the year, including the local Preparation for Professional Practice week, were more useful than those received for later rotations. Longer inductions were more useful than shorter. Departmental inductions were generally only moderately helpful and they missed many important characteristics. Gaps in their inductions identified by many trainees matched those aspects judged to be most useful by those trainees who had experienced these characteristics.

**Conclusions:**

Many Foundation trainee doctors are experiencing inadequate inductions, notably at the department level. Trainees are starting rotations in new departments without rudimentary knowledge about their role and responsibilities in that department, where to find equipment and documentation, who to contact and how to contact them, local preferences, policies and procedures. Unsurprisingly, trainees who do receive such information in their inductions regard it as highly useful. Action is urgently needed to improve departmental inductions so that all trainees have the information they require to work confidently and competently in each new department they rotate into.

**Electronic supplementary material:**

The online version of this article (doi:10.1186/s12909-015-0395-1) contains supplementary material, which is available to authorized users.

## Background

On graduating medical school in the United Kingdom (UK) newly qualified doctors progress to the Foundation Programme. The Foundation Programme is a two-year training programme which builds on undergraduate education. Trainee doctors work on supervised clinical placements (commonly three placements of four months in length each year), rotating through a number of different specialities. Trainee doctors work to consolidate skills learnt in medical school and further improve their performance.

The transition from medical student to trainee doctor can be stressful, and there is evidence that trainee doctors feel unprepared for many aspects of their new role [1–3]. This stress can be worsened for trainee doctors starting a new placement who are faced with an inadequate induction, including a lack of information about their role [1, 4]. Kilminster et al. [5] explored the operationalisation of transitions, including that between medical student and the first Foundation year. They argued that performance is dependent on the characteristics of the specific setting, and that factors such as rotas and induction can hamper performance at times of transition. The value of an appropriate and thorough induction at the start of trainee doctors’ new posts has been well established; it has been shown to improve both confidence and competence [6–8].

When considering the impact of both undergraduate medical school and postgraduate medical training on preparedness, Goldacre et al. [9] argue that a balance needs to be found “between what is taught in medical school, of immediate relevance to the first job, and what is taught as induction at work in the first job” (p8). Trainee doctors themselves acknowledge that there are some components of their role that can only be learnt through experience in that role [2]. Illing et al. [10] commented that trainee doctors need to become familiar with the practical, day-to-day aspects of ward work, some of which “requires knowledge about local logistics – for example, location of the forms for ordering tests and the correct procedures for ordering an x-ray.” (p8). Thus the importance of local induction during Foundation training is evident.

The Foundation Programme requires both a local induction at the start of the first year, including information about the programme and how it will be provided by the particular Foundation School the trainee doctors is registered with, and also clinical induction sessions at the start of each placement [11]. However, data from the National Training Surveys indicate that less than two-thirds of Foundation trainees rated their induction as good or excellent (65.4 % in 2011; 63.1 % in 2013) [12, 13]. Previous research investigating the induction experiences of first year Foundation trainees at one particular Foundation School in detail found that a significant number of trainees were not receiving any induction at all, and that content judged to be useful by trainees was not included in the inductions [8]. Similarly, focus groups conducted with first year Foundation trainee doctors to discuss how prepared they felt during the early weeks of work identified the importance of induction experiences in supporting the transition from medical school to work [14]. Induction content such as shadowing the outgoing Foundation doctor and life support training were highly valued, but the departmental inductions at the start of each clinical placement were lacking in important content which the trainees need to function on a daily basis. The trainees lacked information about processes such as electronic discharging of patients, requesting investigations and the bleep system. They also required information about where to find things in their department, the various teams they would be dealing with, consultant preferences for how they should work, being on call and procedures at night, who to contact for help and, at a very basic level, what their responsibilities as a first year Foundation doctor actually were in the department.

The aim of the reported research was to examine the experiences of trainee doctors’ registered with the East Anglian Foundation School (EAFS) with the various induction programmes on offer at their employing National Health Service (NHS) Trust. It was part of a larger study investigating preparedness to practice of first year Foundation doctors.

## Methods

### 1) Audit questionnaire

The research was carried out at 13 local hospitals (12 NHS Trusts) in the East of England region employing trainee doctors registered with the EAFS. A questionnaire was designed with the purpose of auditing existing Trust induction procedures for first year Foundation doctors (F1s). This Foundation School has a mandatory week long Preparation for Professional Practice (PfPP) period before the August start date for all new F1s which is comprised of formal teaching and shadowing of the outgoing F1 for their new post; so the audit questionnaire covered the Trust induction, PfPP and departmental inductions for the clinical rotations. Specifically, the questions covered: the number of F1s that had started at the Trust August 2010, what induction programmes were on offer, the timing and length of the programmes, F1 attendance at the programmes (whether attendance was mandatory and percentage attendance), whether any of the induction programmes included shadowing an outgoing F1, whether there was a Trust handbook, who runs the programmes, and the content of the Trust induction. This questionnaire was emailed to the postgraduate medical education offices at each participating Trust at the start of the 2010–11 academic year. Twelve of the 13 hospitals returned a completed questionnaire. The audit results were used to refine the questionnaire for clarity, where needed, to conduct a further audit for the 2011–12 academic year and to develop a questionnaire for F1s asking about their induction experiences during that same 2011–12 academic year. Only the 2011–12 data are reported here.

### 2) F1s’ induction experiences questionnaire

A two-part questionnaire was developed for completion by F1s to explore (i) self-perceived preparedness to practice in a number of areas by undergraduate medical training (data not reported) and (ii) experience with inductions programmes on offer at their employing Trust. As noted previously, the questionnaire was designed following the audit of 2010–11 induction programmes. Additionally, the questionnaire design was influenced by the findings of focus groups and interviews with F1s during that same 2010–11 year about their early weeks of work, which included discussion about their induction experiences [14]. Questionnaire data were collected on two occasions, once during the first of the three rotations of the first Foundation year (Rotation 1) and once during the final rotation (Rotation 3). This enabled information to be gathered about initial induction experiences shortly after the F1s started their new roles, and also experiences as they moved to new departments and sometimes different hospitals through changing rotations during the year.

In the induction half of the questionnaire, which is the focus of this paper, F1s were asked to provide details of their current Trust and department; in the Rotation 3 questionnaire they were also asked to provide these details for their previous two rotations. They were then asked to indicate the length of their induction for their current department and the usefulness of the departmental handbook and various elements of that induction (18 items). F1s were asked to rate the helpfulness of all the inductions they had experienced during their first Foundation year so far for preparing them for their F1 role. Four open ended questions asked the F1s to provide details of:

- the most useful aspects of their Trust / PfPP induction week,
- the most useful aspects of their departmental inductions,
- any gaps in the inductions,
- suggestions to fill these gaps, for improving the induction process.

In the Rotation 3 questionnaire those F1s who had moved Trusts between rotations were asked to comment on their experiences with induction at the new Trust/s. The following demographic data were also collected: gender, age, year of qualification, name of medical school, whether the medical course had been a graduate entry course, and whether the course had included any time shadowing a Foundation doctor (questionnaire available from corresponding author).

### Data collection

The invited sample comprised of all 312 trainee doctors currently employed on F1 posts through the EAFS at 12 NHS Trusts (13 hospitals) in the East of England region. Two recruitment methods were used. One of the authors visited teaching sessions at as many of the Trusts as could be arranged to introduce the study and distribute hard copies of the questionnaire (all 13 hospitals for Rotation 1 and 12 for Rotation 3). F1s were provided the opportunity to complete the questionnaire at that time or complete it later and post it back to the research team using a provided pre-paid envelope. Online completion of the questionnaire was also provided for F1s who had not been in attendance at the teaching session. The EAFS sent the link for the online questionnaire with the participant information sheet attached to all F1s at the start of each phase of data collection, followed by two reminders. The data collection period lasted approximately 2 months to enable the site visits to take place; starting after the F1s had been in post for approximately 4 weeks, to allow them time to settle into their new post and discover what they did and did not know following their induction experiences. For each of the two rotations, participating F1s were provided with the opportunity to be entered into a prize draw to win one of five £50 vouchers from a well-known internet retailer.

### Study design

Whilst the measure of interest (the questionnaire) was repeated on two occasions, the participants were free to complete both or just one of the questionnaires and the questionnaires were anonymous to encourage honest responses. So it was not known which F1s had provided which data, and thus the data were not related. A non-experimental study design was used, where all participants were asked to complete the same questionnaires. Both quantitative and qualitative data were collected through the use of closed and open questions, respectively. The sample was self-selected, in that the participants volunteered to participate by completing a questionnaire that was presented to them.

### Ethics

NHS ethical approval was obtained from the Cambridgeshire 4 Research Ethics Committee (reference number 10/H0305/7), and Research and Development approval was obtained from all the 12 Trusts (Additional file 1). The research was carried out in compliance with the Helsinki Declaration.

### Data analysis

Non-parametric analysis was used, as the data were not normally distributed. The relationship between the length of the departmental induction and how helpful it was judged to be was examined using Spearman’s rho. Chi-square analysis was performed to compare demographic characteristics by the two rotations. All analysis was conducted using SPSS 16.0 for Windows. The open-ended comments were subjected to basic content analysis by two of the authors, with input from an assistant, to categorise and summarise the data. As the questionnaire was in part designed to investigate perceptions of preparedness by medical school training (data not reported) it was decided to exclude participants who graduated from medical school before 2010 from the analysis, to minimise the effect of recall bias.

## Results

### 1) Audit questionnaire

#### Audit of 2011–12 inductions programmes

The audit of induction procedures conducted for the 2011–12 academic year was completed by 11 of the 13 hospitals. At the time of the study, PfPP was up to 7 days in length and took place before the August start date. It included at least 50 % of the time shadowing an outgoing F1. The audit findings indicated that 100 % of the F1s attended PfPP in 9 of the Trusts, 98 % in 1 Trust and 90 % in 1 Trust. All 11 hospitals also had a Trust induction; in 8 of the hospitals this was integrated into PfPP and it was separate in 3 hospitals. All Trusts had a Trust handbook that was distributed to F1 doctors as part of the induction process, this varied in format, including hard copy (7 hospitals), online (4 hospitals), CD (3 hospitals); 2 hospitals indicated that the handbook was available in more than one format. Only 1 hospital offered shadowing for F1s at the start of their second and third rotations. Ten of the 11 hospitals provided details about the induction experiences on offer to F1 doctors joining from another Trust for their second or third rotation; this generally comprised of the standard Trust induction only.

The results of the audit indicated that all Trusts included the following in their induction procedures: Blood Transfusion training, Clinical governance, Fire health and safety, Infection control, IT training, Prescribing skills, Risk management, consent and complaints, Where to access clinical guidelines and protocols, Resuscitation training, Sickness, and Requesting leave & leave allowance. Ten of the 11 hospitals covered the E-portfolio during induction, and the other included it in the first formal F1 teaching session. Thus this content was covered by all hospitals in the early days of the F1s first rotation. Eight to 10 of the hospitals also covered: Career support, Child protection, Equality and Diversity, Hospital at Night, Hospital security, Incident reporting, Locum arrangements, Manual handling, Occupational health, Pharmacy, Review of practical skills.

### 2) F1s’ induction experiences questionnaire

#### Sample characteristics

213 F1s completed the questionnaire in Rotation 1 (153 completed in the teaching session, 13 returned by post, 47 completed online) and 185 completed it in Rotation 3 (143 in the teaching session, 7 by post, 35 online). Questionnaires were received from F1s working at all of the 13 hospitals in both rotations. After removing the data from F1s who had graduated before 2010 and from participants who did not fully complete the questionnaire the final numbers were 192 for Rotation 1 (62 % response rate) and 165 (53 % response rate) for Rotation 3 (357 in total); 92 % of whom had graduated in 2011 in both rotations (the rest in 2010).

The demographic background information for each rotation separately can be found in Table 1. The medical school curricula were classified as traditional if there was a clear pre-clinical/clinical divide in the programme; reformed if there was a substantial degree of vertical integration of the programme; and PBL where the main learning method throughout the course was problem-based learning. There were no significant differences between the two rotations for any of these areas.

**Table 1:** Background information for each rotation

Participant background information		Rotation 1 First survey	Rotation 3 s survey
		N = 192	N = 165
Gender	Male	80 (42 %)	60 (36 %)
	Female	110 (57 %)	103 (62 %)
Age	Mean (SD)	26.26 (3.90)	26.99 (4.05)
	Range	23 to 42	23 to 53
Graduate entry course	Yes	34 (18 %)	29 (18 %)
	No	155 (81 %)	132 (80 %)
Shadowing during medical training	Yes	176 (92 %)	152 (92 %)
	No	7 (4 %)	11 (7 %)
Medical School Type	Traditional	66 (34 %)	41 (25 %)
	PBL	78 (41 %)	81 (49 %)
	Reformed	41 (21 %)	35 (21 %)
Location of Medical School	UK	184 (96 %)	153 (93 %)
	Overseas	6 (3 %)	8 (5 %)

NB Some participants did not answer all the background questions; percentages reported are in relation to the full responding sample, so percentages reported in the table add up to less than 100 %

### F1s’ inductions experiences

F1s were asked to rate how helpful their various inductions had been in preparing them for their F1 role on a 7 point scale (where 1 indicated that the induction was “No help at all”, and 7 that the induction was “Extremely helpful”). Findings indicated that the PfPP induction programme was regarded as being most helpful (Table 2). Departmental inductions were rated as progressively less helpful throughout the three rotations.

**Table 2:** Helpfulness of inductions

Induction	Mean (SD)
Preparation for Professional Practice (PfPP)	5.01 (1.46)^a^
Trust induction	4.60 (1.44)^a^
Rotation 1 Departmental induction	4.73 (1.54)^a^
Rotation 2 Departmental induction	4.16 (1.71)^b^
Rotation 3 Departmental induction	4.04 (1.61)^b^

Data from: ^a^Rotation 1, ^b^Rotation 3

F1s were asked to outline the most useful aspects of the PfPP/Trust inductions they had experienced, the following were highlighted:

- Shadowing the job they would be doing
- Time with, and talks from, outgoing F1 doctors
- Meeting other staff in the department and other new F1s
- Being given local knowledge - learning about local policies, paperwork / forms, learning their way around the hospital
- Being told how to make referrals and request investigations
- Being told who to contact, when and how to contact them
- Training on, and getting access to, the computer systems
- Information and training on dealing with emergencies
- Revising clinical skills and how to use equipment (e.g. defibrillator)

F1s were also asked to provide details of the most useful aspects of any of the departmental inductions they had experienced during the F1 year, the following areas were mentioned:

- Being introduced to the full team/department, including the consultant, and being told who does what
- Being given an explanation of what was expected of them as an F1, including their daily duties and responsibilities, their role in the team, and preferences for how tasks are done
- Having the rota explained to them and finding out what they are expected to do on the different shifts
- Being told how to contact different people, including how to contact senior staff for support.
- Being given a tour of the department and being told where things are
- Being given a handbook
- Being told how to request investigations
- Meeting with, or being given information from, the outgoing F1

The F1s were asked to indicate the length of the departmental induction they had received for their current placement. Data from the Rotation 1 and 3 questionnaires were combined as participants were rating different departmental inductions in each questionnaire. The findings indicated that the length of the departmental induction varied widely (Table 3), with half being less than or equal to 2 h. A small number of F1s had received no departmental induction at all.

**Table 3:** Length of departmental induction in Rotations 1 and 3

Length	Number of F1s (percentage)
Less than 1 h	103 (29 %)
1 to ≤2 h	74 (21 %)
>2 h to ≤4 h	25 (7 %)
>4 h to 1 day	47 (13 %)
More than 1 day	61 (17 %)
Induction not provided	28 (8 %)
Did not attend induction	6 (2 %)

Correlation analysis, using Spearman’s rho, was conducted to investigate if there was any relationship between the length of the departmental induction and its perceived helpfulness. A significant correlation of 0.424 (significant at 0.01) was found, indicating that as length of induction increased so did its helpfulness.

The majority of those F1s who had had a departmental handbook said that it was useful (86 %), but 42 % of the F1s had not had a departmental handbook at all.

The F1s were asked to indicate how useful they had found 18 potential components of the departmental induction using three categories: “Not at all Useful”, “Moderately Useful” and “Extremely Useful”; a “Not covered” option was also available. Again, as different departmental inductions were being rated by different participants, the results from Rotations 1 and 3 were combined.

All 18 components were judged to be Extremely Useful by at least a third of the F1s who answered these questions. Over 50 % rated the following components of their departmental induction to be Extremely Useful:

- How to order tests and related paperwork
- Senior support and how to them contact in day
- Discharge policies and related paperwork
- Being given a tour of department and being shown where everything is
- Being told about how to deal with cardiac arrest and where the trolley is
- Senior support and how to them contact at night
- The teaching sessions, programme, your requirement to attend
- What is expected of you as an F1 in the department
- The regular duties of the job

Of these most useful aspects of the departmental inductions, 22 % of the F1s who responded to these questions indicated that “Senior support and how to them contact at night” had not been covered in their departmental induction; 37 % indicated that “Being given a tour of department and being shown where everything is” had not been included in their departmental induction; and 46 % reported that “Being told about how to deal with cardiac arrest and where the trolley is” had not been included in their departmental induction.

Other useful components of the departmental induction that had been missing for a fifth or more of the participants who answered the departmental induction questions included “Consultant preferences for patient management” (46 %), “Referral policies” (38 %), “Who to contact in different departments” (36 %), “Study leave policies” (27 %), “Handover policies” (25 %), and “Being introduced to key members of the department” (20 %).

When asked via an open ended question what gaps the F1s felt there had been across all of the induction experiences available to them the following areas were highlighted:

- Not having received a departmental induction at all
- Not having ID badges, passwords, access codes for doors to enable them to do everything expected of them as soon as they started their job
- Not having a tour of the department and/or hospital
- A lack of knowledge about their duties and responsibilities, and what was expected of them
- Not knowing who to contact or how to contact people, particularly senior staff, including out of hours
- Not knowing what is involved in being on-call/working out of hours and lack of explanation about handovers
- Lack of explanation about rotas and annual leave policies

When asked how such gaps could be filled F1s requested a departmental induction, including meeting consultants and other staff, tour and a departmental handbook. More specifically, they wanted more information about their duties and what is expected of them, including on-call/out of hours, and more information about how to do a handover. During the PfPP week, they wanted more time shadowing, including shadowing of on-call/out of hours, talks from the outgoing F1s about day-to-day jobs and how things ‘work’, and for some of the lectures to be revised so as to be more relevant to their needs regarding their day-to-day duties.

In the Rotation 3 questionnaire, those F1s who had moved Trusts during the year were asked to provide details about any problems they had encountered due to lack of adequate induction at their new hospital. There were fewer responses to this question, as it did not apply to many of the participants (as they had stayed at the same Trust for all three rotations). Several participants commented that they had not encountered any problems or spontaneously mentioned that their induction had been good. The main problem encountered related to lack of familiarity with the new computer systems and the related issue of not having the required accesses (e.g. ID badges, passwords) to do what they needed to do at the start of the rotation. A few participants commented that there had been a general lack of information about what was expected of them and that there was no one available to help them and/or they did not know whom to ask. Other participants simply made general comments about not having a departmental induction or having an unspecified poor induction experience.

## Discussion

The findings indicate that F1s are undergoing variable induction experiences. Most F1s report that PfPP, the week-long induction provided by the EAFS immediately prior to the August start date, is helpful. Comments indicated that components of this induction programme, including shadowing the outgoing F1 and formal teaching such as revision of clinical skills, are valued by the F1s. This is in line with the findings of focus groups conducted with F1s the previous year at the same 12 Trusts [14]. These F1s also described the usefulness of PfPP, in particular shadowing in the post they were about to start. However, they did point out that this usefulness was dependent on both the time of day at which the shadowing took place (in relation to when valuable ward activities were taking place) and the willingness of the outgoing F1 to engage with them and find useful activities for them to undertake.

In contrast to the generally positive PfPP induction experience, the F1s’ experience with the departmental inductions is more variable, particularly in later rotations. The findings from the questionnaires suggest that longer departmental inductions are more useful, but half of the F1s experienced inductions of only 2 h or less, and some did not have a departmental induction at all. Similarly there are multiple components to the departmental inductions judged to be useful by F1s, which many other F1s are not experiencing. These findings are consistent with the earlier focus group study [14] and support those of Thomson et al. [8] who, investigating the induction experiences of F1s at another UK foundation school, found that F1s were not receiving many useful topics in their departmental inductions.

The F1s are facing many important gaps in their induction experiences. At the most basic level, many have not been told their duties, responsibilities or what is expected of them. Additionally, they have not been given a tour of the department, so they do not know where anything is. In some cases F1s are unable to do their job when they start a new rotation because they have not been provided with identification badges, passwords for computer systems or access codes for doors to secure areas. They also do not know what is involved in being on-call/working out of hours, how the rotas and handovers at shift changes work, or the annual leave policies. They do not know who to contact or how to contact them, including accessing senior support out-of-hours. Additionally, the audit of inductions indicated that F1s rotating into a new Trust for their second or third rotation generally only receive the standard Trust induction, which does not usually include those aspects that the F1s rated as useful in a departmental induction or the valued components of PfPP such as shadowing the new rotation’s post. As a result many F1s are dealing with considerable uncertainty about rudimentary aspects of their F1 role, which previous research indicates represents an unnecessary additional stressor during this time of transition between medical school and work as a trainee doctor [1, 4].

The induction programme serves a number of purposes. At the most basic level it provides a new employee with information relating to employment matters such as rotas, annual leave procedures, health and safety issues and Trust policies on a variety of topics. This induction is commonly generic and applies to all grades of staff. The findings suggest that most of the Trust inductions addressed these areas. More significantly, for the new F1, the induction programme provides a useful bridge between medical school and clinical practice. If it is effective it should mitigate the anxiety caused by the step change in responsibility that individuals experience during this transition [14]. The PfPP programme appears to go some way towards providing this support. A third purpose for the induction programme is ensuring patient safety, as newly qualified doctors begin to undertake patient care. This component should be addressed in the departmental induction. While it is clear that there are some departments that provide examples of good practice others are falling short. This appears to be more of a problem in the second and especially in the third rotations, when possibly it is assumed that the F1 is now experienced and does not need any further orientation. Familiarity with the physical geography of the department; an awareness of the acceptable procedures and protocols; and above all a clear understanding of when and how clinical problems should be escalated to senior members of staff are all fundamental to ensuring patient safety. It is concerning that a substantial number of respondents here stated that these areas had not been addressed in their departmental inductions.

Thomson et al. [8] recommended that departmental inductions follow a standardised format containing a minimum level of detail; that the standard and content of inductions are monitored; and that F1s are involved in determining the content of the inductions. The findings of this research support these recommendations. It is evident that F1s have clear thoughts about what they need to know in order to optimally function in their F1 role. Ensuring that all departmental inductions cover this same key information, with flexibility for additional department-specific topics, will likely lead to more useful induction experiences for F1s, and guarantee that all of their rotations to different specialities are equally as well supported. Thought should be given to the nature and duration of the induction and all departments should be required to provide a comprehensive handbook for junior staff.

It is recognised that making quality improvements to departmental inductions will not be an easy task; it is possible that in some Trusts this will not be amenable to central control. As such, the postgraduate medical education offices will be reliant on multiple individuals to determine the department-specific topics necessary for their incoming F1s to know and also to deliver all the agreed-on content. However, in considering the use of clinical rotations in both undergraduate and postgraduate medical education, Holmboe et al. [15] commented that the multiple transitions trainee doctors experience via their clinical rotations can negatively impact on socialisation into the medical profession, relationships with patients, team-working competencies and opportunities for assessment and feedback with seniors. Thus, it has to be considered that any adverse effects of repeatedly changing teams, specialities and locations will be aggravated by a poor induction experience, making it all the more vital that Trusts review their departmental inductions in light of findings such as those reported here and make a concerted effort to improve F1s induction experiences.

Whilst F1s from 13 different hospitals were involved in the induction experience questionnaire and the audit involved data from 11 of these hospitals, the key limitation of this study is that it was conducted within the context of a single Foundation School. Whilst the findings reported here do support those of Thomson et al. [8], who conducted their study in a different Foundation School, it is unknown how generalizable the findings might be to other Foundation Schools. Additionally, this particular Foundation school has a structured week-long induction period (the PfPP programme) prior to the commencement of the first Foundation year, which is designed to provide the opportunity to have early questions answered and offer key preparatory experiences to support the F1s in their transition from medical student to junior doctor. The results of this study indicate that this induction experience is regarded as valuable by F1s, and it is unclear whether the useful and relevant content and positive experience of the PfPP programme mitigates the poorer departmental inductions on offer. Thus it is possible that the departmental induction experiences of F1s who have not had this initial Foundation School-wide induction experience will differ from the reported findings. However, it is conceivable that without such early support, F1s in other Foundation Schools may be facing an inferior induction experience overall; which would make the findings of the reported study equally of interest to other postgraduate medical education centres looking to improve the induction experiences they offer to incoming F1s.

## Conclusions

In conclusion, trainee doctors need to be supported by adequate and appropriate inductions, to ensure that they are optimally prepared for the specific experience of each clinical placement they undertake during their two years on the Foundation Programme. However the findings of this research indicate that induction experiences, particularly at the department level, are currently not sufficient to meet the F1s’ needs. The findings show that the information and support needs of F1s from their inductions are known; now it is necessary to ensure that all departmental inductions are fully inclusive of this content.

## Additional file

Additional file 1:
**Trust Research and Development committees/departments who gave permission for the research to be conducted.**
