# Supplementary material for: Foundation doctors’ induction experiences
Source: BMC Med Educ. 2015 Jul 24;15:118. doi: 10.1186/s12909-015-0395-1 (PMC4513374; doi:10.1186/s12909-015-0395-1)
Supplement: Additional file 1: — Trust Research and Development committees/departments who gave permission for the research to be conducted. [file 12909_2015_395_MOESM1_ESM.docx]

**Appendix 1**

Trust Research and Development committees / departments who gave permission for the research to be conducted

| **Name of Trust** |
| --- |
| Bedford Hospital NHS Trust |
| Cambridge University Hospitals NHS Foundation Trust (for Addenbrookes Hospital NHS Trust) |
| Hertfordshire Hospitals R&D Consortium (for Lister Hospital & the Queen Elizabeth II Hospital) |
| Hinchingbrooke Health Care NHS Trust |
| James Paget University Hospitals NHS Foundation Trust |
| Luton and Dunstable Hospital NHS Foundation Trust |
| Norfolk and Norwich University Hospitals NHS Foundation Trust |
| Papworth Hospital NHS Foundation Trust |
| Peterborough Hospitals NHS Trust |
| The Ipswich Hospital NHS Trust |
| The Queen Elizabeth Hospital King’s Lynn NHS Trust |
| West Suffolk Hospital NHS Trust |
